# Forage and breed effects on behavior and temperament of pregnant beef heifers

**DOI:** 10.1186/2049-1891-4-20

**Published:** 2013-05-28

**Authors:** Angela R Mays, Michael L Looper, Benjamin C Williamson, Kenneth P Coffey, Wayne K Coblentz, Glen E Aiken, Charles F Rosenkrans

**Affiliations:** 1 Department of Animal Science, University of Arkansas, Fayetteville, AR, 72701, USA; 2 USDA-ARS, US Dairy Forage Research Center, Marshfield, WI, 54449, USA; 3 USDA-ARS, Forage Animal Production Research Unit, Lexington, KY, 40546, USA; 4 Department of Agricultural Sciences, Morehead State University, 327 Reed Hall, Morehead, KY, 40351, USA

**Keywords:** Cattle, Endophyte-infected tall fescue, Grazing behavior, Prolactin, Temperament

## Abstract

**Background:**

Integration of behavioral observations with traditional selection schemes may lead to enhanced animal well-being and more profitable forage-based cattle production systems. Brahman-influenced (BR; n = 64) and Gelbvieh × Angus (GA; n = 64) heifers consumed either toxic endophyte-infected tall fescue (E+) or one of two nontoxic endophyte-infected tall fescue (NT) cultivars during two yr. Heifers were weighed at midpoint and termination of grazing. Grazing behavior (grazing, resting in the shade, lying, or standing without grazing) was recorded (n = 13 visual observations per yr in June and July) for each pasture. During yr 2, exit velocity (EV) and serum prolactin (PRL) were determined.

**Results:**

Grazing behavior was influenced (*P* < 0.05) by an interaction between fescue cultivar and breed type. Gelbvieh × Angus heifers assigned to E+ pastures had the lowest percentage of animals grazing and the largest percentage of animals resting in the shade. Brahman-influenced heifers had faster EV (*P* < 0.001) than GA heifers (0.52 vs. 0.74 ± 0.04 s/m, respectively). Body weight (BW) was affected (*P* < 0.01) by an interaction of tall fescue cultivar and d, and an interaction of tall fescue cultivar and breed type. Heifers grazing NT pastures were heavier (*P* < 0.01) than heifers grazing E+ pastures at midpoint and termination. Gelbvieh × Angus heifers grazing NT pastures were heavier (*P* < 0.01) than GA and BR heifers grazing E+ and BR heifers grazing NT pastures. An interaction of forage cultivar and breed type occurred on serum PRL (*P* < 0.01).

**Conclusion:**

Collectively fescue cultivar, EV, and concentrations of serum PRL were associated with grazing behavior. Heifers grazing NT pastures were observed to be grazing more than heifers assigned to E+ pastures, regardless of breed type, which may have contributed to changes in BW and average daily gain (ADG) in heifers. Integration of behavioral observations along with traditional selection schemes may lead to enhanced animal well-being and more profitable forage-based cattle production systems.

## Background

Tall fescue [*Lolium arundinaceum* (Schreb.) Darbysh.] is a highly adaptive grass species with over 16 million ha found in the southern and eastern regions of the USA [1]. Ergot alkaloids, such as ergovaline, are produced by an endophytic fungus (*Neotyphodium coenophialum*) that infects tall fescue plants [2]. Consumption of E+ leads to fescue toxicosis, which is characterized by reduced feed intake and ADG, elevated body temperature, increased respiration rate, and poor conception rates [3-6]. Health issues associated with E+ are estimated to cost livestock producers over $1 billion annually [7].

Cattle response to E+ consumption has been related to breed composition with Brahman-influenced cattle being more tolerant of ergot alkaloids, resulting in improved weight gain and reproductive success while grazing E+ when compared to British breeds of cattle [8-10]. Tall fescue cultivars infected with nontoxic novel endophytes do not produce ergot alkaloids and lead to faster livestock gains compared to animal gains when grazing E+ [6,11]. Cultivars of NT have plant persistence similar to E+ with animal performance equivalent to endophyte-free tall fescue [12]. While animal performance of cattle grazing E+ and NT is well documented, it is unknown if differential animal response is due to physiological responses or behavioral and temperament changes. Cattle with excitable temperaments exhibit reduced weight gain, and milk production [13,14]. Therefore, our objective was to evaluate the effects of tall fescue cultivar and breed type on weight gain, behavior, temperament, and concentrations of serum prolactin (PRL) of pregnant beef heifers.

## Methods

### Experimental design

Research was conducted at USDA-Agriculture Research Service, Dale Bumpers Small Farms Research Center, Booneville, Arkansas, USA (35°09’N, 93°17’W). Animal procedures used for this 2-yr study were approved by the USDA-Agriculture Research Service animal welfare committee. Treatments were arranged as a 2 × 3 factorial with main effects of breed and tall fescue cultivar. Heifers (18 ± 2 mo of age) were either Brahman-influenced (1/8 to 1/3 *Bos indicus*; BR) or Gelbvieh × Angus (GA). Tall fescue cultivars were E+, and nontoxic [Jesup infected with strain AR542 endophyte (MQ; 13), or HiMag with strain 4 endophyte (HM; 6)]. Year 1 consisted of 72 heifers (36 of each breed) grazing from 28 March until 5 July. During yr 2, 56 heifers (28 of each breed) began grazing 29 March and continued until 18 July. On d 0 of both yr, heifers were weighed and randomly assigned within breed type to graze tall fescue pastures (4 heifers/ha). Year 1 consisted of six pastures per fescue cultivar while yr 2 consisted of four pastures of E+, five pastures of HM and five pastures of MQ. Stocking rate was four heifers per hectare (2 heifers per breed). All pastures were established >3yr and were >85% fescue; pastures were not allowed to go to seed. Water was provided *ad libitum*, forage was not limited during the study, and shade was available for all animals. Heifers were weighed on d 56 (midpoint) and 99 (termination of grazing) in yr 1, and on d 60 (midpoint) and 116 (termination of grazing) during yr 2. To assess toxicity of fescue pastures, random forage samples (8 to 10 samples per ha) were collected monthly from each pasture, pooled within pasture, cut into 5.1-cm pieces, and stored at −4°C until ergovaline concentrations were determined using high-performance liquid chromatography [15].

### Behavior data

Grazing behavior was visually recorded in yr 1 and 2 between 13:00 and 15:30 h on 13 dates, in individual pastures, during the months of June and July (n = 13 visual observations). Within yr, each heifer (n = 4 heifers per pasture) was observed visually by the same technicians at a distance of 75 to 100 m for approximately 2 min. Heifer behavior was classified into one of four categories (grazing, resting in shade, lying, or standing without grazing). During yr 2, chute exit velocity (EV), an indicator of animal temperament, was determined on d 0, 60, and 116 using two infrared sensors (FarmTek Inc., North Wiley, Texas, USA). Heifers were gathered with all-terrain four-wheeled vehicles on each data collection day by the same personnel and walked from individual pastures 305 to 1,240 m to individual holding pens; heifers remained in their individual pasture group during the handling process. Heifers were moved from pens to a curved working alley (0.8 m wide and 7.9 m in length) with solid sides. Body weight was recorded and a blood sample collected while each heifer was restrained in the hydraulic handling chute (Filson Livestock Equipment, Protection, Kansas, USA). As heifers exited the handling chute and traversed 1.8 m, EV (s/m) was recorded [16].

Maximum ambient daily temperature and maximum daily relative humidity (%) during visual observation of grazing behavior were recorded using a weather station (model 900, Spectrum Technologies Inc., Plainfield, Illinois, USA) located 5 km from the tall fescue pastures.

### Blood collection and hormone analysis

In yr 2, blood samples were obtained by venipuncture of the median caudal vein into vacuum tubes (Becton, Dickinson, Franklin Lakes, New Jersey, USA) on d 0, 60, and 116. Blood samples were allowed to clot for 24 h at 4°C and centrifuged (1,500 × *g* for 25 min). Serum was stored at −4°C until PRL concentrations were analyzed by radioimmunoassay [17], with intra-assay coefficient of variation (CV) of 11% and inter-assay CV of 15%.

### Statistical analysis

Weight gain and behavioral variables did not differ (*P* > 0.10) between heifers consuming the two NT cultivars (HM or MQ); therefore, means and percentages for NT treatments were pooled. Body weight, ADG, PRL, and EV data were analyzed using mixed model procedures of SAS (SAS Inst., Inc., Cary NC) with pasture as the experimental unit. Model assumed a completely randomized block design with pasture as experimental unit. Fescue cultivar and breed type were replicated within yr and across yr. Fixed effects of fescue cultivar (E+ vs. NT), breed type (BR vs. GA), and interactions were compared using F-test protected t-tests (*P* < 0.05). Fescue cultivar, breed type, and interactive effects on observed grazing behavior were analyzed by Chi-square.

## Results

### Forage and environmental conditions

Concentrations of ergovaline ranged from 0.25 to 0.87 mg/kg of dry matter (pooled SD = 0.3) for E+ tall fescue pastures from April to mid-July; overall mean ergovaline was 0.55 ± 0.2 mg/kg of dry matter. Forage samples were pooled prior to determination of ergovaline. Without replicated values, statistical analysis on concentration of ergovaline in E+ tall fescue pastures was not conducted. No detectable ergovaline was found in NT pastures. On day when behavior was observed, maximum ambient daily temperature averaged 31.1 ± 2.6°C and mean maximum relative humidity was 98.2 ± 3.7%.

### Grazing behavior

**Figure 1:**
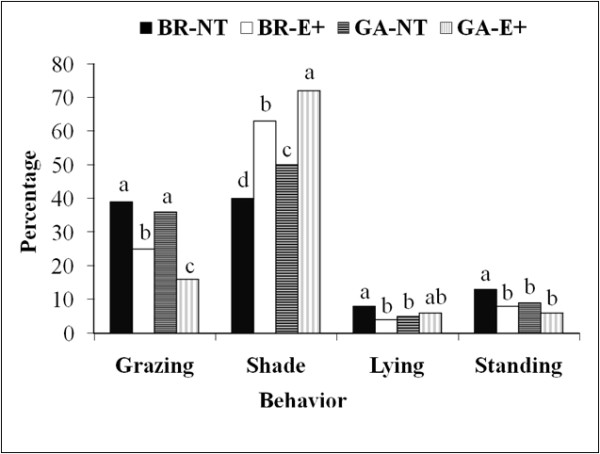
**Interaction of forage cultivar and breed type on grazing behavior of beef heifers; **^**abcd**^***P < *0.05.**

Grazing behavior between 13:00 and 15:30 h was influenced (*P* < 0.05) by an interaction between fescue cultivar and breed type (Figure 1). Gelbvieh × Angus heifers assigned to E+ pastures had the lowest (*P* < 0.05) percentage of animals classified as grazing and the largest (*P* < 0.05) percentage of animals resting in the shade. In contrast, heifers assigned to NT pastures had the largest percentage of animals classified as grazing and BR heifers assigned to NT pastures had the lowest percentage resting in the shade.

### Prolactin concentrations

**Figure 2:**
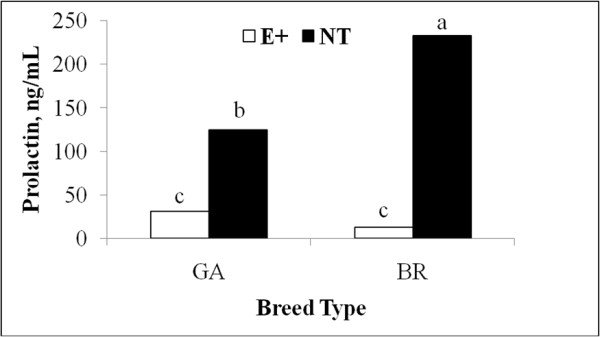
**Interaction of forage cultivar and breed type on serum prolactin of beef heifers; **^**abc**^***P < *0**.**05; pooled SE = 21.25.**

Serum PRL concentrations were affected (*P* < 0.05) by an interaction of fescue cultivar and breed type (Figure 2). Brahman-influenced heifers grazing NT pastures had greater (*P* < 0.05) PRL concentrations than GA heifers grazing NT pastures (233.2 ± 16.5 vs. 125.2 ± 16.5 ng/mL), and BR and GA heifers grazing E+ had the lowest (30.8 ± 26 and 13.5 ± 20 ng/mL; *P* < 0.05) concentrations of PRL.

### Exit velocity

Brahman-influenced heifers traversed the 1.8 m distance faster (*P* < 0.001) than GA heifers (0.52 vs. 0.74 ± 0.04 s/m, respectively). Exit velocity also was influenced by d of collection (*P* < 0.02) with EV on d 0 not different (*P* > 0.06) than EV on d 60 or d 116 (0.61, 0.54, and 0.73 ± 0.05 s/m, respectively). However, EV on d 60 was faster (*P* < 0.01) than EV on d 116. Forage cultivar did not influence (*P* > 0.10) exit velocity throughout the study.

### Body weight and average daily gain

**Figure 3:**
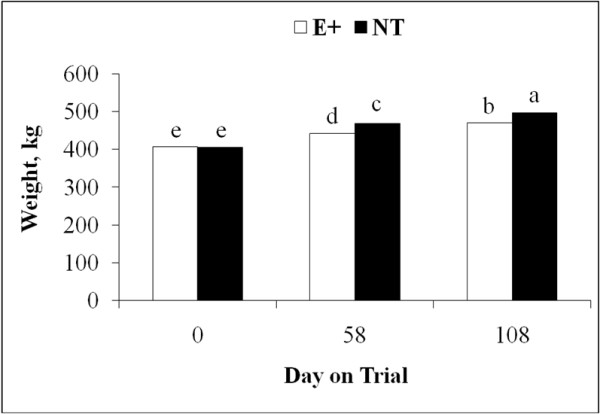
**Interaction of forage cultivar and day on body weight of beef heifers; **^**abcde**^***P < *0**.**01; pooled SE = 4.9.**

**Figure 4:**
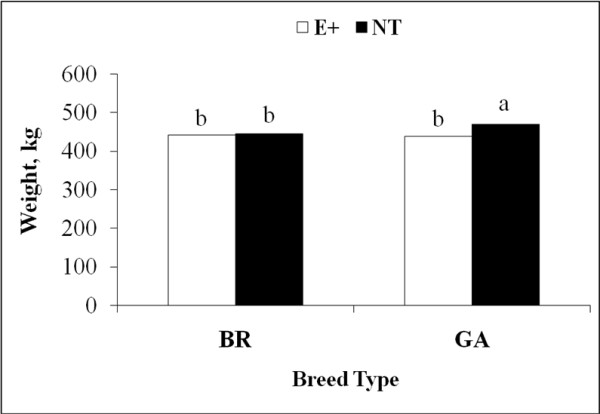
**Interaction of forage cultivar and breed type on body weight of beef heifers; **^**ab**^***P < *0.05; pooled SE = 4.0.**

Body weight was affected (*P* < 0.01) by an interaction of fescue cultivar and day of collection, and an interaction of fescue cultivar and breed type. Body weights at the initiation of grazing were not different (407 vs. 405 ± 5 kg; *P* > 0.10) among heifers assigned to E+ and NT pastures. Heifers grazing NT pastures were heavier (*P* < 0.01) than heifers grazing E+ pastures at midpoint (58 ± 3 d) and termination (108 ± 12 d) of grazing (Figure 3). Gelbvieh × Angus heifers grazing NT pastures were heavier (*P* < 0.01) than GA and BR heifers grazing E+ and BR heifers grazing NT pastures (Figure 4).

Heifer ADG was calculated for each period (first period = d 0 to midpoint; second period = midpoint to termination) of the study and overall ADG. During the first period (58 ± 3 d), heifer rate of gain was faster (*P* < 0.0001) on NT pastures when compared with heifers grazing E+ pastures (1.1 vs. 0.6 ± 0.04 kg). Gelbvieh × Angus heifers had faster rate of gain (*P* < 0.0001) than BR heifers (0.96 vs. 0.8 ± 0.04 kg). In contrast, ADG during the second period (50 ± 9 d) was not affected (*P* > 0.11) by fescue cultivar, breed type, or their interaction. During the 108 ± 12 d study, overall heifer ADG was affected by breed type and fescue cultivar. Rate of gain by GA heifers was faster (*P* < 0.02) than that of BR heifers (0.77 vs. 0.69 ± 0.03 kg). Heifers grazing NT pastures exhibited greater (*P* < 0.0001) ADG than heifers grazing E+ pastures (0.87 vs. 0.60 ± 0.03 kg).

## Discussion

Fescue toxicosis is a condition that occurs in livestock and can alter grazing behavior [18,19]. Ergovaline is the primary ergopeptine found in E+ tall fescue, which leads to physiological changes and results in fescue toxicity [20,21]. Our E+ pastures had ergovaline concentrations (0.55 mg/kg of dry matter) during June and July that were similar to published values of ergovaline capable of inducing fescue toxicosis in cattle and sheep [22,23]. Dietary concentrations of ergovaline between 0.400 and 0.750 mg/kg DM have been reported to cause fescue toxicosis in cattle [24].

Pregnant heifers have an elevated body metabolism and fasting heat production [25]. That increased demand for body temperature regulation coupled with the stress associated with E+ led us to study breed type and fescue cultivar on grazing behavior of pregnant beef heifers. We observed that GA heifers assigned to E+ pastures had a lower percentage of heifers grazing during midday compared with BR heifers grazing E+ pastures. Heifers grazing MQ and HM nontoxic endophyte-infected pastures, in the current study, were observed to be grazing more than heifers assigned to E+ pastures, regardless of breed type. In a previous study steers spent more time grazing endophyte-free tall fescue compared with E+, and steers consuming E+ were more sensitive to solar radiation [26].

Cattle consuming E+ typically have reduced PRL concentrations [5,7]. Heifers grazing E+ had reduced PRL concentrations as compared to heifers grazing NT pastures in the present study, and PRL concentrations of heifers on E+ were not influenced by breed type. The BR heifers grazing NT pastures had the greatest percentage of animals grazing and increased PRL concentrations; those findings support the possible connection between PRL and animal behavior. Increased activity, which occurs during grazing, was associated with increased PRL concentrations [27]. The mechanism describing the physiological and behavioral relationship between activity and PRL concentrations is not yet fully understood.

In this study, temperament of heifers may have contributed to changes in BW and ADG. Cattle had faster EV times at the initiation of grazing but EV times slowed as the study progressed. These data support previously reported research in which temperament of animals improved over time, presumably due to adaptation of repeated handling [16]. Gelbvieh × Angus heifers had slower EV times suggesting better temperaments relative to BR heifers, which is consistent with previous work reporting more docile tempered animals had greater BW and ADG [13,28].

## Conclusions

Collectively fescue cultivar, EV, and serum PRL concentrations were associated with grazing behavior, which may contribute to changes in BW and ADG. Integration of behavioral observations along with traditional selection schemes may lead to enhanced animal well-being and more profitable forage-based cattle production systems.

## Abbreviations

BR: Brahman-influenced; GA: Gelbvieh × Angus; E+: Toxic endophyte-infected tall fescue; NT: Nontoxic endophyte-infected tall fescue; PRL: Prolactin; ADG: Average daily gain; EV: Exit velocity; CV: Coefficient of variation

## Competing interests

The authors declare that they have no competing interests.

## Authors’ contributions

MLL conceived the study and experimental design. ARM, CFR, and MLL carried out the statistical analysis and drafting of manuscript. BCW compiled data and revised initial draft manuscript. KPC and WKC supplied the experimental animals and revised manuscript. GEA established and managed pastures used in study. All authors read and approved the final manuscript.
